# The difficulty of protein structure alignment under the RMSD

**DOI:** 10.1186/1748-7188-8-1

**Published:** 2013-01-04

**Authors:** Shuai Cheng Li

**Affiliations:** 1 Department of Computer Science, City University of Hong Kong, Kowloon, Hong Kong

**Keywords:** Protein Structure, Alignment, RMSD, LCP

## Abstract

**Background:**

Protein structure alignment is often modeled as the *largest common point set* (LCP) problem based on the Root Mean Square Deviation (RMSD), a measure commonly used to evaluate structural similarity. In the problem, each residue is represented by the coordinate of the C*α*atom, and a structure is modeled as a sequence of 3D points. Out of two such sequences, one is to find two equal-sized subsequences of the maximum length, and a bijection between the points of the subsequences which gives an RMSD within a given threshold. The problem is considered to be difficult in terms of time complexity, but the reasons for its difficulty is not well-understood. Improving this time complexity is considered important in protein structure prediction and structural comparison, where the task of comparing very numerous structures is commonly encountered.

**Results:**

To study why the LCP problem is difficult, we define a natural variant of the problem, called the *minimum aligned distance* (MAD). In the MAD problem, the length of the subsequences to obtain is specified in the input; and instead of fulfilling a threshold, the RMSD between the points of the two subsequences is to be minimized. Our results show that the difficulty of the two problems does not lie solely in the combinatorial complexity of finding the optimal subsequences, or in the task of superimposing the structures. By placing a limit on the distance between consecutive points, and assuming that the points are specified as integral values, we show that both problems are equally difficult, in the sense that they are reducible to each other. In this case, both problems can be exactly solved in polynomial time, although the time complexity remains high.

**Conclusions:**

We showed insights and techniques which we hope will lead to practical algorithms for the LCP problem for protein structures. The study identified two important factors in the problem’s complexity: (1) The lack of a limit in the distance between the consecutive points of a structure; (2) The arbitrariness of the precision allowed in the input values. Both issues are of little practical concern for the purpose of protein structure alignment. When these factors are removed, the LCP problem is as hard as that of minimizing the RMSD (MAD problem), and can be solved exactly in polynomial time.

## Background

A common approach to understand the properties of a protein is to compare it to other proteins. Proteins that are similar, in terms of either their amino acid sequences or 3-dimensional structures, often share similar functions, or are related evolutionarily. The latter, structural comparison, is particularly interesting since protein structures are known to be more evolutionarily conserved than the biological sequences which encode them. Furthermore, proteins of similar structures may have similar functionality, even when their sequences differ [1].

Structural comparison is typically a problem of aligning two sets of 3-dimensional coordinates. (In most of the known structural alignment problems, each point is the 3D coordinates of the C*α* atom, one per residue. Hence, a structure can be modeled for structural alignment purpose as a sequence of 3D points.) The alignment usually involves a rigid transformation to superimpose the two sequences of points, and a mapping which specifies the matched points. The parameters to optimize in the alignment may differ in different situations, because it is not easy to single out a set of parameters that best captures the similarity between two given structures [2]. In many situations, the alignment needs not match between every point in the two sequences. At present, there is a consensus among molecular biologists in the use of the following two parameters [2-4]: 

1. the number of residues (points) or percentage of total residues (points) matched in the alignment.

2. the root mean square deviation (RMSD) of the matched residues (points).

In general, the RMSD need not be minimized. It suffices that it is within a reasonable threshold. Hence, a good alignment is customarily taken to be one which maximizes the number of residue matches, within a given RMSD threshold. Many structural alignment methods are based on this principle. The computational complexity of finding an optimal solution to the problem is not well understood. Shibuya *et al.* formulated a restricted version of the problem, and showed the problem to NP-hard when the dimensionality is arbitrary. It is open whether their problem is NP-hard in 3-dimension [5]. Other problems related to structural comparison based on the RMSD have been found to be difficult. For example, the problem of finding a substructure from multiple 3-dimensional structures which minimizes the total RMSD, is NP-hard [6].

For the variants of the alignment problem that are not based on the RMSD, we have the following results. When the objective is to maximize the number of point matches which are no more than a threshold distance apart, the problem is solvable in *O*(*n*^32.5^) time, where *n* is the number of points [7]. The *contact map overlap* problem, where a graph is created out of each structure, and the problem is one of comparing the two graphs, is NP-hard [8], and remains NP-hard even when we require points that are matchable to be within a threshold distance [9]. These results, together with an early result which shows a related problem called threading to be NP-hard [10], have traditionally led molecular biologists to believe that the structural alignment problem is difficult in general (e.g. [11-13]), even though a PTAS exists for the problem under a broad class of distance measures [14]. Heuristic algorithms have also been proposed for many variants of structural alignment problem [15-23]. While these methods perform reasonably well in general, they provide no guarantee on the quality of their results.

As noted by Shibuya *et al.*, relatively few theoretical results have been obtained on problems defined over the RMSD, and the general problem of structural alignment under the RMSD remains open [5]. At present, whether the problem is intractable or not is not only of theoretical interests but also of practical concerns, due to advances in protein structure prediction which requires the comparison of very numerous structures. In this paper we show mathematical insights and techniques which we hope will lead to practical algorithms for the problem.

We first show that the difficulty of the problem does not lie solely in the individual components of their requirement. More precisely, then the problem can be solved in polynomial time.

– if either a mapping that contains the optimal mapping is known (Theorem 3), or

– if the optimal superposition is known (Lemma 1),

Our study shows that the difficulty of the LCP problem is also very much due to the two factors: (1) the problem allows the input coordinates to be of any arbitrary precision, and (2) it assumes no limit on the distance between two consecutive C*α*atoms.

We consider the case where the input coordinates are integral, and the distance between two consecutive points is restricted. The first requirement is practical since in protein structures, coordinates are typically specified to a fixed precision (e.g. three decimal places in protein structures [24]), and can be trivially scaled up to integral values. Similar assumptions are made in Euclidean problems such as the Euclidean TSP [25]. The second requirement likewise does not add any restriction to the problem of protein structure alignment, since there is a natural upper bound (∼3.8Å) to the distance between two C*α*atoms. In this case, the following results hold. 

– Given a polynomial time algorithm for finding a largest alignment of RMSD below a threshold *d*, one can efficiently compute an alignment of a given size *ℓ* which minimizes the RMSD (Theorem 7). (Since the other direction is easy, this shows that the two problems are of similar difficulty.)

– The structural alignment problem under the RMSD is solvable exactly in polynomial time (Theorem 10).

## Preliminary

### Notations and definitions

A protein structure for alignment purpose is modeled as a finite, ordered sequence of three dimensional (3D) points. Hence, a structure of *n* residues is written as (*p*_1_,*p*_2_,*...*,*p*_*n*_), where each point $p_{i}\;\in \;R^{3}.$In the ‘Results assuming integral coordinates and restricting distance between points’ section, we will further assume each *p*_*i*_ to be integral. We write *P*^′^ ⊆ *P* iff *P*^′^ is a subsequence of *P*, and write *f*:*P* ↦ *Q* iff *f* is a mapping which maps points in the sequence *P* to points in the sequence *Q*.

### Problem statements

We now state our problems. The main problem we consider is the largest common point set (LCP) problem under the RMSD, a well-known problem in protein structure alignment. In the LCP, the objective is to find a mapping of the largest cardinality where the RMSD of the matched points is no more than a given threshold (Table 1).

**Table 1:** Largest Common Point (LCP) set problem definition under RMSD

	**LCP problem by the RMSD measure**
**Input:**	sequences *P* = (*p*_1_,…,*p*_*n*_), *Q* = (*q*_1_,…,*q*_*m*_) and distance
	threshold θ∈R. Without loss of generality assume *m* ≥ *n*.
**Output:**	(i) subsequences *P*^′^ ⊆ *P*, *Q*^′^ ⊆ *Q*, |*P*^′^| = |*Q*^′^|, and
	(ii) bijection *f*:*P*^′^ ↦ *Q*^′^, fulfilling the following conditions:
	(A) *RMSD*(*P*^′^,*f*(*P*^′^)) ≤ *θ*,
	(B) the score *l* = |*P*^′^| is maximized.

We do not require the optimal superposition of *P* and *Q* in the output, since that can be computed from *P*^′^, *Q*^′^, and *f* in linear time [26]. We refer to *f* as an *alignment*. An alignment can be *sequential* or *non-sequential*: an alignment is *sequential* iff for any two points $p_{i_{1}}$, $p_{i_{2}}\in P^{\prime }$, where the corresponding $f(p_{i_{1}})=q_{j_{1}}$ and $f(p_{i_{2}})=q_{j_{2}}$, we have *i*_1_*i*_2_ iff *j*_1_*j*_2_. Otherwise the alignment is *non-sequential*. The LCP problem which requires alignments to be sequential is said to be *sequential*, otherwise it is *non-sequential*. We mainly discuss sequential alignment in this paper. The techniques developed can be easily adapted to the non-sequential case. Given two equal-sized sequences *P*^′^ = (*p*_1_,…,*p*_*n*_) and *Q*^′^, together with a bijection *f* between *P*^′^and *Q*^′^, the root mean square deviation (RMSD) is defined as

(1) $$\mathrm{RMSD}(P^{\prime },Q^{\prime })=\underset{\text{t}}{\min}\sqrt{\frac{\underset{1\leq i\leq n}{\sum }||\text{t}(f(p_{i}))-p_{i}|{|}^{2}}{n}},$$

where *t* is a rigid transformation. The RMSD, with its corresponding transformation *t*, can be computed in linear time [26].

A natural variant of the LCP problem is to minimize the RMSD instead of maximizing the size of the mapping, as follows. Given an integer *ℓ*, find subsequences of size *ℓ* of the input, such that the RMSD between the points of the subsequences is minimized. We call this problem the minimum aligned distance (MAD) problem (Table 2).

**Table 2:** Minimum Aligned Distance (MAD) problem definition under RMSD

	**MAD problem by the RMSD measure**
**Input:**	sequences *P* = (*p*_1_,…,*p*_*n*_), *Q* = (*q*_1_,…,*q*_*m*_) and ℓ∈I.
	Without loss of generality assume *m* ≥ *n*.
**Output:**	(i) subsequences *P*^′^ ⊆ *P*, *Q*^′^ ⊆ *Q*, |*P*^′^| = |*Q*^′^|, and
	(ii) bijection *f*:*P*^′^ ↦ *Q*^′^, fulfilling the following conditions:
	(A) |*P*^′^| = *ℓ*,
	(B) *d* = *RMSD*(*P*^′^,*f*(*p*^′^)) is minimized.

Clearly, if the MAD problem is solvable in polynomial time, then the LCP problem is solvable in polynomial time. However, the other direction is unclear. Theorem 7 will show that for *P* and *Q* of integral coordinates, if the LCP problem is solvable in polynomial time, then the MAD problem is solvable in polynomial time.

We let $\hat{P}$, $\hat{Q}$, $\hat{f}$, *ℓ* and *d*_*opt*_ denote an optimal *P*^′^, *Q*^′^, *f*, *l* and *d*, respectively. The optimal rigid transformation for superimposing $\hat{P}$ and $\hat{Q}$ is denoted T, and can be computed from $\hat{P}$, $\hat{Q}$ and $\hat{f}$. The symbol $c_{P}^{\text{max}}$ denotes the largest value in the coordinates of *P*, $c_{Q}^{\text{max}}$ the largest value in the coordinates of *Q*, and $c_{\text{max}}=\text{max}\{c_{P}^{\text{max}},c_{Q}^{\text{max}}\}$, and we know that *c*_*max*_ = *O*(*n*) for protein structures.

## Results for general LCP and MAD

### Complexity of the LCP and MAD when the optimal superposition is known

Since two point sequences with a known mapping can be superimposed optimally under the RMSD in linear time [26], it is natural to ask if the difficulty in LCP or MAD lies solely in the combinatorial complexity of finding the optimal subsets, i.e. $\hat{P}$ and $\hat{Q}$. Our results show the contrary: if the optimal superposition T is known, both problems can be solved in polynomial time.

We first consider the sequential case. Let *d*_*p*,*q*_ = ||*t*(*p*) − *q*||^2^ and let *M*[*i*,*j*;*k*] denote the minimum squared sum cost of *k* pair matches for the point sets (*p*_1_,*p*_2_,*...*,*p*_*i*_) and (*q*_1_,*q*_2_,*...*,*q*_*j*_). If 1 ≤ *k* ≤ *ℓ*, 2 ≤ *i* ≤ *m* and 2 ≤ *j* ≤ *n*, we have a recurrence relation of

(2) $$M[i,j;k]=\text{max}\left\{\begin{array}{l} M[i-1,j-1;k-1]+d_{p_{i},q_{j}},\\ M[i,j-1;k],\\ M[i-1,j;k] \end{array}\right\}$$

The base case of the recursion is obvious. Dynamic programming can be employed to fill up the respective *M*[*i*,*j*;*k*] values. After all the values are filled, one can find the maximum *k*, such that the squared sum is no more than *kθ*^2^ for the LCP problem. The MAD problem can be solved similarly.

The non-sequential case can be similarly solved using the maximum-flow minimum-cost problem [27]. The following lemma states these results.

#### Lemma 1

If an optimal transformation T is known, both the LCP problem and the MAD problem can be solved in *O*(*mnℓ*) time.

### Complexity of the LCP and MAD when the matching between the point sets is known

We next ask if the difficulty in the LCP and MAD could be due to the task of superimposing *P* and *Q* in an optimal manner to identify the subsets $\hat{P}$ and $\hat{Q}$. To examine this possibility, we remove the combinatorial task of examining each of the possible mapping between the points, by assuming a bijection *F* which contains the optimal mapping. (Note that this results in the problem known as *model superposition* in structural biology.) Again, our results show the resultant LCP and MAD problems to be solvable exactly in polynomial time.

Assume that *F* is a bijection that maps points in *P* to points in *Q*. Let $P^{\prime }=(p_{1}^{\prime },\ldots ,p_{l}^{\prime })$ be the domain of *F* and $Q^{\prime }=(q_{1}^{\prime },\ldots ,q_{l}^{\prime })$ be the range of *F*. Without loss of generality assume that *F* is sequential, and hence $F(p_{i}^{\prime })=q_{i}^{\prime }$.

One can exhaustively evaluate all the subsequences of *P*^′^ for one with the least RMSD. However, since the number of such subsequences is exponential in *l*, this does not immediately give us a polynomial time solution.

If a rigid transformation *T* for *Q*^′^ is given, and all the pairs $\left(p_{i}^{\prime },q_{i}^{\prime }\right)$, 1 ≤ *i* ≤ *l* are sorted according to the value $\left||T(p_{i}^{\prime })-q_{i}^{\prime }|\right|$, the MAD problem is then to choose the first *ℓ* pairs from $\left(p_{i}^{\prime },q_{i}^{\prime }\right)$, 1 ≤ *i* ≤ *l*, and the LCP problem is to choose the first *ℓ* pairs, such that $\text{RMSD}((p_{1}^{\prime },\mathrm{...},p_{\ell }^{\prime }),(q_{1}^{\prime },\mathrm{...},q_{\ell }^{\prime }))\leq \theta$ and *ℓ* = *l* or $\mathrm{RMSD}((p_{1}^{\prime },\mathrm{...},p_{\ell }^{\prime },p_{\ell +1}^{\prime }),(q_{1}^{\prime },\mathrm{...},q_{\ell }^{\prime },q_{\ell +1}^{\prime }))>\theta$. This gives us an incentive to obtain a total ordering of $\left||T(p_{i}^{\prime })-q_{i}^{\prime }|\right|$, which will allow us to solve the MAD problem by selecting the first *ℓ*pairs in the ordering. The set of transformations which produce the same total according to $\left||T(p_{i}^{\prime })-q_{i}^{\prime }|\right|$, yield the same result for the MAD problem, and therefore these transformations are equivalent. This enables us to design a discrete version of the problem.

For clarity, we first present an algorithm with only translation.

#### With translations only

Consider two pairs $\left(p_{i}^{\prime },q_{i}^{\prime }\right)$ and $\left(p_{j}^{\prime },q_{j}^{\prime }\right)$. The transformations *T* to separate the two types of transformations that $\left||T_{1}(q_{i}^{\prime })-p_{i}^{\prime }||>||T_{1}(p_{j}^{\prime })-q_{j}^{\prime }|\right|$ and $\left||T_{2}(q_{i}^{\prime })-p_{i}^{\prime }||||T_{2}(p_{j}^{\prime })-q_{j}^{\prime }|\right|$ are the transformations where

(3) $$||T(q_{i}^{\prime })-p_{i}^{\prime }|{|}^{2}-||T(p_{j}^{\prime })-q_{j}^{\prime }|{|}^{2}=0.$$

Let ∙denote dot product. If the transformation is a translation *t*, we have

(4) $$\begin{array}{cc} & ||T(q_{i}^{\prime })-p_{i}^{\prime }|{|}^{2}-||T(q_{j}^{\prime })-p_{j}^{\prime }|{|}^{2}\\ = & ||q_{i}^{\prime }-p_{i}^{\prime }-t|{|}^{2}-||q_{j}^{\prime }-p_{j}^{\prime }-t|{|}^{2}\\ = & \underset{v=x,y,z}{\sum }{\left(v_{q_{i}^{\prime }}-v_{p_{i}^{\prime }}-v_{t}\right)}^{2}-\underset{v=x,y,z}{\sum }{\left(v_{q_{j}^{\prime }}-v_{p_{j}^{\prime }}-v_{t}\right)}^{2}\\ = & ||q_{i}^{\prime }-p_{i}^{\prime }|{|}^{2}-||q_{j}^{\prime }-p_{j}^{\prime }|{|}^{2}\\ & -2t∙((q_{i}^{\prime }-p_{i}^{\prime })-(q_{j}^{\prime }-p_{j}^{\prime }))=0 \end{array}$$

Consider the space of all translation vectors in $R^{3}$, and consider each vector as a point in this space (not the space that *P* and *Q* are in). The values that the variable *t* in Equation 4 may take form a plane in this translation space. The plane partitions the translation space into two types of translations, *T*_1_ and *T*_2_ say, where $\left||T_{1}(q_{i}^{\prime })-p_{i}^{\prime }||>||T_{1}(p_{j}^{\prime })-q_{j}^{\prime }|\right|$ and $\left||T_{2}(q_{i}^{\prime })-p_{i}^{\prime }||||T_{2}(p_{j}^{\prime })-q_{j}^{\prime }|\right|$. Since there are *l* pairs, there are *O*(*l*) planes, which partition the space into *O*(*l*^3^) cells.

The translations in each cell result in the same ordering of the pairs with respect to $\left||T(p_{i}^{\prime })-q_{i}^{\prime }|\right|$. For each cell, this total order can be obtained in *O*(*l*) time from any given total order of its neighbor cells, since the change is *O*(1). Therefore, the MAD solution can be obtained in amortized time *O*(*l*) for each cell, and the LCP solutions thus can be obtained in time *O*(*l*^2^). Hence the total runtime is of *O*(*ℓ*^4^) for the MAD problem, and *O*(*ℓ*^5^) for the LCP problem of translations, with the given mapping *F*.

#### With rigid transformations

The rigid transformations which separate the two relations $\left||T_{1}(q_{i}^{\prime })-p_{i}^{\prime }||>||T_{1}(p_{j}^{\prime })-q_{j}^{\prime }|\right|$ and $\left||T_{2}(q_{i}^{\prime })-p_{i}^{\prime }||||T_{2}(p_{j}^{\prime })-q_{j}^{\prime }|\right|$ are as in Equation 3.

Suppose the rigid transformations *T* is composed of a rotation *R* and a translation *t*.

(5) $$\begin{array}{cc} & ||T(q_{i}^{\prime })-p_{i}^{\prime }|{|}^{2}-||T(q_{j}^{\prime })-p_{j}^{\prime }|{|}^{2}\\ = & ||R(q_{i}^{\prime })-p_{i}^{\prime }-t|{|}^{2}-||R(q_{j}^{\prime })-p_{j}^{\prime }-t|{|}^{2}\\ = & ||R(q_{i}^{\prime })-p_{i}^{\prime }|{|}^{2}-||R(q_{j}^{\prime })-p_{j}^{\prime }|{|}^{2}\\ & -2t∙[(R(q_{i})-p_{i})-(R(q_{j})-p_{j})] \end{array}$$

A rotation matrix contains three variables, which is specified using three angles, say *α*_1_, *α*_2_, *α*_3_, each from −*Π* to *Π*. Let *r*_*i*_ = cos *α*_*i*_, *s*_*i*_ = cos *α*_*i*_, then Equation 5 can be considered as a polynomial of nine variables in degree six. The nine variables are *r*_*i*_, *s*_*i*_ and the three variables for translation. In total, there are *O*(*l*) such polynomials.

We know the following theorem from the literature.

##### Theorem 2

[7,28]*Given a set of k polynomials,*$P=\{f_{1},\mathrm{...},f_{k}\}$, *where each polynomial has a maximum degree of s*, *contains at most r variables, and in addition all the coefficients are rational, then all the sign conditions can be determined by O*(*k*(*k*/*r*)^*r*^*s*^*O*(*r*)^)* arithmetic operations. A sign condition V is the vector of signs for some point*$u\in R^{k}$; *that is, V* = (*sign*(*f*_1_(*u*)),*...*,*sign*(*f*_*k*_(*u*))). *Two points*$u,u^{\prime }\in R^{k}$*are equivalent if their sign condition vectors are the same.*

Each sign vector represents the transformations of the cell it belongs to, and it determines a total order of the pairs. Similar as in the case of translation, with Theorem 2, we have

##### Theorem 3

*Given a bijection F*:*P*^′^ → *Q*^′^, *where* |*P*^′^| = *l*, *then the MAD problem can be solved in O*(*l*^10^) *time and the LCP problem can be solved in O*(*l*^11^) *time.*

## Results assuming integral coordinates and restricting distance between points

One possible contributing factor to the difficulty of the LCP problem could be its flexibility in allowing input coordinates of any arbitrary precision. This is because intuitively, this arbitrariness in the precision introduces the burden of examining the solution space in an unbounded manner. However, such an exhaustive search is not necessary for the purpose of protein structure comparison, since coordinates of protein structures are specified only to three decimal places in the commonly used PDB format.

In this section, we restrict the precision in which the input coordinates may be specified. Without loss of generality, we assume that the input coordinates are given in integers, since numbers of any fixed precision can be trivially scaled up to integral values. This assumption is used to obtain Lemma 5 and Theorem 7.

We also place an upper bound on the distance between consecutive points according to the structure of proteins. As a result, *c*_*max*_ is bounded by *n*, as follows.

Points drawn from protein structures have upper bounds on their diameters because they are connected, and many are globular. 

- For a connected structure, the points are at most *O*(*n*) distance apart. That is, *c*_*max*_ is of *O*(*n*).

- For a globular structure, the points are at most *O*(*n*^1/3^) distance apart [14]. That is, *c*_*max*_ is of *O*(*n*^1/3^).

Given a point *p*, let the *x* coordinate of *p* be denoted *x*_*p*_. Similarly, we can define *y*_*p*_ and *z*_*p*_. Without loss of generality, we assume that the first point of a protein structure is at the origin. The largest coordinate of a protein is bounded by *O*(*n*), and the largest coordinate of a globular protein structure is bounded by *O*(*n*^1/3^); that is

(6) $$\underset{p\in P,v=x,y,z}{\text{max}}|v_{p}|=O(n),\text{if P is a protein structure}$$

(7) $$\;\underset{p\in P,v\;=\;x,y,z}{\text{max}}|v_{p}|\;=\;O(n^{1/3}),\text{if P is a globular protein structure}$$

Our results show that, under these two conditions, 

1. the LCP problem is of similar difficulty as the MAD problem, and

2. both problems can be solved exactly in polynomial time.

### Properties of protein structures

#### Upper and lower bounds of RMSD

We first establish some bounds to the RMSD. The minimum RMSD is zero if T brings $\hat{Q}$ to coincide exactly with $\hat{P}$. This case is referred to as the exact matching, which can be easily solved by the method in [29]. However, if we assume the RMSD to be non-zero, then a lower bound and an upper bound for it can be computed.

Let *Π* be a permutation of {1,*...*,*ℓ*}. For the sequence *X* = (*x*_1_,…,*x*_*n*_), let $d_{i,j}^{X}$1 ≤ *i*,*j* ≤ *n*, denote the Euclidean distance between *x*_*i*_ and *x*_*j*_. The following results, which are proven in the Appendix, can be obtained.

##### Lemma 4

$$\begin{array}{c} \frac{1}{\sqrt{2\ell }}\sqrt{\overset{\left\lfloor \ell /2\right\rfloor }{\underset{i=1}{\sum }}|d_{\Pi (i),\Pi (i+\left\lfloor \ell /2\right\rfloor }^{\hat{P}})-d_{\Pi (i),\Pi (i+\left\lfloor \ell /2)\right\rfloor }^{\hat{Q}}{|}^{2}}\\ \;\mathrm{RMSD}(\hat{P},\hat{Q}). \end{array}$$

##### Lemma 5 (**Lower bound**)

*If RMSD*$(\hat{P},\hat{Q})\neq 0$, *then RMSD*$(\hat{P},\hat{Q})\geq \frac{\sqrt{12c_{\text{max}}^{2}}-\sqrt{12c_{\text{max}}^{2}-1}}{\sqrt{2\ell }}$.

##### Lemma 6 (**Upper bound**)

*RMSD*$(\hat{P},\hat{Q})\leq 4\sqrt{3}c_{\text{max}}$.

#### Using an algorithm for the LCP to solve the MAD problem

Suppose there is a polynomial time algorithm for solving the LCP problem. To use it to solve the MAD problem, we assume that *d*_*opt*_ ∈ [*l*,*u*], for some real *l* and *u*, *l* ≤ *u*. We use a binary search strategy in the interval [*l*,*u*], as shown in Table 3, to search for the minimum value such that the LCP solution size is *ℓ*. However, the search will not terminate if an arbitrary accuracy of the *d*_*opt*_ value is required. We prove below that the accuracy of *d*_*opt*_ can be defined by polynomially many bits. Given two threshold *t*_1_ and *t*_2_, assume that we obtain two different LCP solutions, and the RMSD values of the two solutions are *θ*_1_ and *θ*_2_, where *θ*_1_ > *θ*_2_. Similar to the arguments in Lemma 5, the difference between *θ*_1_ and *θ*_2_ is at least $\frac{\sqrt{12c_{\text{max}}^{2}}-\sqrt{12c_{\text{max}}^{2}-1}}{\sqrt{2\ell }}$. Therefore if two consecutive binary search operators have the difference of the threshold values below $\frac{\sqrt{12c_{\text{max}}^{2}}-\sqrt{12c_{\text{max}}^{2}-1}}{\sqrt{2\ell }}$, the search can be terminated. The values of *l* and *u* are the same as in the previous subsection. Hence,

**Table 3:** Employing an algorithm for the LCP problem to solve the MAD problem

**Input:**	sequences *P* = (*p*_1_,…,*p*_*n*_), *Q* = (*q*_1_,…,*q*_*m*_) and ℓ∈I.
	Without loss of generality assume *m* ≥ *n*.
**Output:**	(i) subsequences *P*^′^ ⊆ *P*, *Q*^′^ ⊆ *Q*, |*P*^′^| = |*Q*^′^|, and
	(ii) mapping *f*:*P*^′^ ↦ *Q*^′^, fulfilling the following conditions:
	(A) |*P*^′^| = *ℓ*,
	(B) *d* = *RMSD*(*P*^′^,*f*(*p*^′^)) is minimized.
1.	*l* ← 0, *u* ← *ℓc*_*max*_
2.	*m* ← 1/2(*l* + *u*)
3.	Call LCP to solve the instance (*P*,*Q*,*m*).
4.	If the LCP solution has size no less than *ℓ*
	*u* ← *m*
	else
	*l* ← *m*
5.	If $u-l\leq \frac{\sqrt{12c_{\text{max}}^{2}}-\sqrt{12c_{\text{max}}^{2}-1}}{\sqrt{2\ell }}$,
	Output the most recent LCP solution of size no less than *ℓ*.
	Otherwise, repeat Steps 2-5.

##### Theorem 7

*Solving the MAD problem is equivalent to solving O*(log*ℓc*_*max*_) *instances of the LCP problem.*

Since the reduction from the LCP problem to the MAD problem is obvious, we conclude that the two problems are of similar difficulty.

### Polynomial time algorithm

We now show that under the two conditions, the LCP and MAD problem can be solved in polynomial time.

As shown in the ‘Complexity of the LCP and MAD when the optimal superposition is known’ section, when the optimal superposition is known, there are polynomial time algorithms for LCP and MAD. We consider an enumeration of all the possible superpositions. Under the two conditions, we claim that there are at most polynomially many such superpositions.

First, if we know $\hat{P}$ and $\hat{Q}$, then optimal superposition can be computed in the following two steps: 

1. Translate $\hat{P}$ and $\hat{Q}$ such that their centroids are at the origin.

2. Then, rotate $\hat{Q}$ to find the superposition with the minimum distance [26].

Denote the translations to obtain the optimal solution for *P* and *Q* as *t*_*P*_ and *t*_*Q*_, respectively, and denote the optimal rotation by $\hat{R}$.

We now show that one needs to examine only polynomially many translations and rotation combinations to discover the values for *t*_*P*_, *t*_*Q*_, and $\hat{R}$. These numbers can be effectively bounded by *n* when properties of protein structures are taken into account. We first describe these properties.

#### Number of translations

The centroid of $\hat{P}$ is $\frac{\underset{p^{\prime }\in \hat{P}}{\sum }p^{\prime }}{\ell }$. To bring $\hat{P}$ to origin, the translation is $-\frac{\underset{p^{\prime }\in \hat{P}}{\sum }p^{\prime }}{\ell }$. Clearly, all the three coordinates of $-\underset{p^{\prime }\in \hat{P}}{\sum }p^{\prime }$ are integers since all the coordinates of the points in *p* ∈ *P* are integers. The value of *x*-coordinate of $-\frac{\underset{p^{\prime }\in \hat{P}}{\sum }p^{\prime }}{\ell }$ is bounded by $-\frac{\underset{p^{\prime }\in \hat{P}}{\sum }x_{p^{\prime }}}{\ell }\leq \frac{\underset{p^{\prime }\in \hat{P}}{\sum }c_{P}^{\text{max}}}{\ell }\leq c_{\text{max}}$. Similarly, all the three coordinates of the translation $-\frac{\underset{p^{\prime }\in \hat{P}}{\sum }p^{\prime }}{\ell }$ are bounded within the interval [−*c*_*max*_,*c*_*max*_]. To obtain an optimal MAD solution, the translation on $\hat{P}$ must be in the form of $\frac{I}{\ell }$, where *I* is an integer. Since it is possible to examine all the possible values for *I*, we have the following result.

##### Lemma 8

*t*_*P*_,*t*_*Q*_ ∈ {*I*/*ℓ*| − *ℓc*_*max*_ ≤ *I* ≤ *ℓc*_*max*_}^3^.

#### Number of rotations

With the centroids of $\hat{P}$ and $\hat{Q}$ translated to the origin, we proceed to identify the rotation in our algorithm. Let *X*_*P*_ denote the vector $\langle x_{p_{1}},\mathrm{...},x_{p_{\ell }}\rangle$ for structure *P*. Similarly we define *Y*_*P*_ and *Z*_*P*_.

Let ${\hat{P}}_{t}=(p_{1}^{\prime }-t,\mathrm{...},p_{\ell }^{\prime }-t)$ and ${\hat{Q}}_{t}=(q_{1}^{\prime }-t,\mathrm{...},q_{\ell }^{\prime }-t)$, $p_{i}^{\prime }\in \hat{P}$, $q_{i}^{\prime }\in \hat{Q}$.

Given $\hat{P}$ and $\hat{Q}$, to compute the rotation $\hat{R}$, the first step is to create the 3 × 3 matrix, which is (from [26])

(8) $$M=\left(\begin{array}{lll} X_{{\hat{P}}_{{\text{t}}_{P}}}∙X_{{\hat{Q}}_{{\text{t}}_{Q}}} & X_{{\hat{P}}_{{\text{t}}_{P}}}∙Y_{{\hat{Q}}_{{\text{t}}_{Q}}} & X_{{\hat{P}}_{{\text{t}}_{P}}}∙Z_{{\hat{Q}}_{{\text{t}}_{Q}}}\\ Y_{{\hat{P}}_{{\text{t}}_{P}}}∙X_{{\hat{Q}}_{{\text{t}}_{Q}}} & Y_{{\hat{P}}_{{\text{t}}_{P}}}∙Y_{{\hat{Q}}_{{\text{t}}_{Q}}} & Y_{{\hat{P}}_{{\text{t}}_{P}}}∙Z_{{\hat{Q}}_{{\text{t}}_{Q}}}\\ Z_{{\hat{P}}_{{\text{t}}_{P}}}∙X_{{\hat{Q}}_{{\text{t}}_{Q}}} & Z_{{\hat{P}}_{{\text{t}}_{P}}}∙Y_{{\hat{Q}}_{{\text{t}}_{Q}}} & Z_{{\hat{P}}_{{\text{t}}_{P}}}∙Z_{{\hat{Q}}_{{\text{t}}_{Q}}} \end{array}\right)$$

Each above matrix is decomposed by the singular value decomposition, and a rotation matrix is produced hereafter.

We know that the coordinate of each point in the protein is within the interval [−*c*_*max*_,*c*_*max*_]. This implies that for *U* = *X*,*Y*,*Z*and *V* = *X*,*Y*,*Z*,

$$\begin{array}{cc} \;U_{{\hat{P}}_{{\text{t}}_{P}}}∙V_{{\hat{Q}}_{{\text{t}}_{Q}}} & =\overset{\ell }{\underset{k=1}{\sum }}(p_{i,k}-{\text{t}}_{P})(q_{j,k}-{\text{t}}_{Q})\leq \overset{\ell }{\underset{k=1}{\sum }}{\left(2c_{\text{max}}\right)}^{2}\\ & \leq 4\ell c_{\text{max}}^{2}. \end{array}$$

Also, it is clear that $U_{{\hat{P}}_{{\text{t}}_{P}}}∙V_{{\hat{Q}}_{{\text{t}}_{Q}}}$ is in the form of *I*/*ℓ*^2^, where *I* is an integer. The matrix in Equation 8 has nine elements; we denote *e* ∈ *M* if *e* is one of these elements. The following lemma follows.

##### Lemma 9

For each element *e* ∈ *M*in Equation 8, $e\in \{I/\ell^{2}|-4\ell^{3}c_{\text{max}}^{2}\leq I\leq 4\ell^{3}c_{\text{max}}^{2}\}$.

#### Polynomial time algorithm

To compute the optimal MAD solution, we first enumerate all the possible translations and rotations. A solution is computed for each translation and rotation combination according to Lemma 1. An optimal solution can be chosen from these computed solutions (Table 4).

**Table 4:** A polynomial time algorithm for the MAD problem

**Input:**	sequences *P* = (*p*_1_,…,*p*_*n*_), *Q* = (*q*_1_,…,*q*_*m*_) and ℓ∈I.
	Without loss of generality assume *m* ≥ *n*.
**Output:**	(i) subsets *P*^′^ ⊆ *P*, *Q*^′^ ⊆ *Q*, |*P*^′^| = |*Q*^′^|, and
	(ii) mapping *f*:*P*^ ′ ^↦*Q*^′^, fulfilling the following conditions:
	(A) |*P*^′^| = *ℓ*,
	(B) *d* = *RMSD*(*P*^′^,*f*(*p*^′^)) is minimized.
1.	For each translation *t* ∈ {*I*/*ℓ*| − *ℓc*_*max*_ ≤ *I* ≤ *ℓc*_*max*_}^3^,
	For each 3 × 3 matrix *M*, where ∀*e* ∈ *M*, *e* ∈ {*I*/*ℓ*^2^|−,
	$\;\{4\ell^{3}c_{\text{max}}^{2}\leq I\leq 4\ell^{3}c_{\text{max}}^{2}\}$
	Compute rotation matrix *R* from *M*.
	Q←RQ−t.
	Apply an algorithm for the case where the superposition
	is known to *P* and Q (as discussed in the ‘Complexity Of
	The LCP And MAD When The Optimal Superposition Is
	Known’ section), and denote the solution MAD(*P*, Q).
2.	Output the MAD(*P*, Q) of the smallest *RMSD* as the solution.

According to Lemma 8, the optimal translation *t*_*P*_ and *t*_*Q*_ must be within {*I*/*ℓ*| − *ℓc*_*max*_ ≤ *I* ≤ *ℓc*_*max*_}^3^. To find the optimal rotation matrix, it suffices that we try all the possible values for each entry in Equation 8. Since there are $\ell^{27}c_{\text{max}}^{18}$ matrices, the number of total transformations to examine is bounded by $O(\ell^{33}c_{\text{max}}^{34})$. It takes time *O*(*mnℓ*) to identify the MAD solution for each transformation. An LCP solution can be obtained by iterating *ℓ* from 1 to min{*m*,*n*} for the MAD problem.

The running time consists of the productions of three parts: the number of possible translations, the number of possible rotation matrix, and the running time for given a rotation matrix and a translation combination (that is, the running time when then transformation is known). These numbers are bounded by *c*_*max*_, which is bounded by *m* when we consider the properties of protein structures. Likewise, *c*_*max*_ is polynomial with respect to the input size if coded in unary.

##### Theorem 10

*The MAD problem can be solved in O*(*ℓ*^34^*m*^25^*n*) *time for protein structures, and in O*(*ℓ*^34^*m*^9^*n*) *time for globular protein structures. The LCP problem can be solved in O*(*ℓ*^35^*m*^25^*n*) *time for protein structures, and in O*(*ℓ*^35^*m*^9^*n*) *time for globular protein structures. Both the MAD and LCP problems are pseudo-polynomially solvable for general point sets.*

## Conclusions

We studied the LCP problem under the RMSD in this paper. As it turns out, the difficulty of the problem does not lie in its combinatoric aspect or its structural superposition aspect alone. That is, if the problem is hard, then it must be a consequence of both aspects. Our results show that if one is allowed to compromise on one of the aspects, then the problem is solvable exactly in polynomial time. Regrettably, we do not see how the optimal solution can be obtained in both cases.

On the other hand, we showed an encouraging result: There is a polynomial time algorithm which solves the problem optimally, if one restricts the input coordinates in the problem to be integral, and places a limit on the distance between consecutive points. These requirements do not pose any restriction to typical uses in the analysis of protein structures, since protein structures are specified only to a fixed precision in practice, and there is an upper bound to the distance between protein residues.

One problem is that our proposed polynomial time algorithm remains high in time complexity. We hope that the present work will provide the foundation for future efforts to obtain algorithms with lower runtime complexities.

## Appendix

In this Appendix, we include the proofs of the results in the paper which have been omitted to enhance readability.

### Lemma 4

$$\begin{array}{cc} \frac{1}{\sqrt{2\ell }} & \sqrt{\overset{\left\lfloor \ell /2\right\rfloor }{\underset{i=1}{\sum }}|d_{\Pi (i),\Pi (i+\left\lfloor \ell /2)\right\rfloor }^{\hat{P}}-d_{\Pi (i),\Pi (i+\left\lfloor \ell /2)\right\rfloor }^{\hat{Q}}{|}^{2}}\\ & \leq \mathrm{RMSD}(\hat{P},\hat{Q}). \end{array}$$

### Proof

Without loss of generality, we just show that

$\frac{1}{\sqrt{2\ell }}\sqrt{\overset{\left\lfloor \ell /2\right\rfloor }{\underset{i=1}{\sum }}|d_{i,i+\left\lfloor \ell /2\right\rfloor }^{\hat{P}}-d_{i,i+\left\lfloor \ell /2\right\rfloor }^{\hat{Q}}{|}^{2}}\leq \mathrm{RMSD}(\hat{P},\hat{Q})$.

Let

$r_{i}=||T(q_{i})-p_{i}|{|}^{2}+||T(q_{i+\left\lfloor \ell /2\right\rfloor })-p_{i+\left\lfloor \ell /2\right\rfloor }|{|}^{2}$,

*u*_*i*_=〈*p*_*i*_,*p*_*i* + ⌊*ℓ*/2⌋_〉, and

*v*_*i*_=〈*q*_*i*_,*q*_*i* + ⌊*ℓ*/2⌋_〉, where 1≤*i*≤⌊*ℓ*/2⌋.

First, we prove that *r*_*i*_ ≥ |*u*_*i*_ − *v*_*i*_|^2^ / 2, for 1 ≤ *i* ≤ ⌊*ℓ*/2⌋. We first superimpose *u*_*i*_ and *v*_*i*_ to optimize the squared sum; that is, to find transformation *T* such that ||*T*(*q*_*i*_)−*p*_*i*_||^2^ + ||*T*(*q*_*i* + ⌊*ℓ*/2⌋_)−*p*_*i* + ⌊*ℓ*/2⌋_||^2^ is minimized. The centroids have to coincide to minimize the squared distance. Assume the centroids are at the origin and that the angle between $\vec{\left(o,p_{i}\right)}$ and $\vec{\left(o,q_{i}\right)}$ is *α*, where *o* is the origin, then by trigonometry

$$\begin{array}{c} ||T(q_{i})-p_{i}|{|}^{2}+||T(q_{i+\left\lfloor \ell /2\right\rfloor })-p_{i+\left\lfloor \ell /2\right\rfloor }|{|}^{2}\\ =2[{\left(1/2\times ||u_{i}||\right)}^{2}+{\left(1/2\times ||v_{i}||\right)}^{2}\\ \;\;\;\;\;\;-2\;\times \;1/2||u_{i}||\;\times \;1/2||v_{i}||\times \cos\alpha ]\\ \geq \frac{{\left(||u_{i}||-||v_{i}||\right)}^{2}}{2} \end{array}$$

*r*_*i*_ is the squared distance under transformation T, which may not be optimal for superimposing *u*_*i*_and *v*_*i*_. Therefore,

$$r_{i}\geq \frac{{\left(||u_{i}||-||v_{i}||\right)}^{2}}{2}$$

Putting things together, we have

$$\begin{array}{cccc} \mathrm{RMSD}(\hat{P},\hat{Q}) & \geq & \sqrt{\frac{r_{1}+\ldots +r_{\left\lfloor \ell /2\right\rfloor }}{\ell }}\\ & \geq & \sqrt{\frac{{\left(u_{1}-v_{1}\right)}^{2}+\ldots +{\left(u_{\left\lfloor \ell /2\right\rfloor }-v_{\left\lfloor \ell /2\right\rfloor }\right)}^{2}}{2\ell }}\\ & \geq & \frac{1}{\sqrt{2\ell }}\sqrt{\overset{\left\lfloor \ell /2\right\rfloor }{\underset{i=1}{\sum }}|d_{i,i+\left\lfloor \ell /2\right\rfloor }^{\hat{P}}-d_{i,i+\left\lfloor \ell /2\right\rfloor }^{\hat{Q}}{|}^{2}} & \end{array}$$

□

### Lemma 5

If RMSD $(\hat{P},\hat{Q})\neq 0$, then RMSD $(\hat{P},\hat{Q})\geq \frac{\sqrt{12c_{\text{max}}^{2}}-\sqrt{12c_{\text{max}}^{2}-1}}{\sqrt{2\ell }}$.

### Proof

If RMSD $\left(\hat{P},\hat{Q}\right)$ is non-zero, then there is at least a pair of indices *i* and *j*, such that $||d_{i,j}^{\hat{P}}$− $d_{i,j}^{\hat{Q}}||>0$. According to Lemma 4,

$$\begin{array}{cc} \mathrm{RMSD}(\hat{P},\hat{Q}) & \geq \frac{1}{\sqrt{2\ell }}\sqrt{|d_{i,j}^{\hat{P}}-d_{i,j}^{\hat{Q}}{|}^{2}}=\frac{\left|d_{i,j}^{\hat{P}}-d_{i,j}^{\hat{Q}}\right|}{\sqrt{2\ell }}\\ & >=\frac{\sqrt{12c_{\text{max}}^{2}}-\sqrt{12c_{\text{max}}^{2}-1}}{\sqrt{2\ell }}. \end{array}$$

                           □

### Lemma 6

RMSD $(\hat{P},\hat{Q})\leq 4\sqrt{3}c_{\text{max}}$.

### Proof

Denote the furthest point to the origin in *P* as *p*_*max*_, and the furthest point to the origin in *Q* as *q*_*max*_. Then,

$$\begin{array}{c} \;\ell \times \mathrm{RMS}D^{2}(\hat{P},\hat{Q})\\ \;=\overset{\ell }{\underset{i=1}{\sum }}||T(q_{i})-p_{i}|{|}^{2}\\ \;\leq \overset{\ell }{\underset{i=1}{\sum }}{\left(||q_{i}-p_{i}||\right)}^{2}\\ \;\leq \overset{\ell }{\underset{i=1}{\sum }}\text{max}\{||q_{\text{max}}-p_{\text{max}}|{|}^{2},||q_{\text{max}}+p_{\text{max}}|{|}^{2}\}\\ \;\leq \ell \text{max}\{||q_{\text{max}}-p_{\text{max}}|{|}^{2},||q_{\text{max}}+p_{\text{max}}|{|}^{2}\}\\ \;\leq \ell {\left(2\sqrt{{\left(c_{\text{max}}\;+\;c_{\text{max}}\right)}^{2}\;+\;{\left(c_{\text{max}}+c_{\text{max}}\right)}^{2}\;+\;{\left(c_{\text{max}}\;+\;c_{\text{max}}\right)}^{2}}\right)}^{2}\\ \;=48\ell c_{\text{max}}^{2} \end{array}$$

                                 □

## Abbreviations

LCP: Largest Common Point set;RMSD: Root Mean Square Deviation

## Competing interests

The authors declare that they have no competing interests.

## Authors’ contributions

SCL conceived of the results herein and wrote the manuscript.
